# The effects of ciprofol on haemodynamics under general anaesthesia during thoracoscopic surgery: a randomised, double-blind, controlled trial

**DOI:** 10.1186/s12871-025-03054-6

**Published:** 2025-04-10

**Authors:** Lifang Lan, Jianping Liao, Liuying Qin, Xuemei Wang, Tan Qin, Yanhua Chen, Jingchen Liu

**Affiliations:** 1 Department of Anesthesiology, Cardiovascular Institute, the First Affiliated Hospital of Guangxi Medical University, Nanning, 530021 Guangxi China; 2 Department of Anesthesiology, Guiyang Maternal and Child Health Care Hospital, Guiyang, 550001 Guizhou China; 3 Department of Anesthesiology, the First Affiliated Hospital of Guangxi Medical University, Nanning, 530021 Guangxi China

**Keywords:** Ciprofol, Propofol, General anaesthesia, Thoracoscopic surgery, Cardiovascular events, Haemodynamic

## Abstract

**Background:**

Propofol, a widely administered sedative, is associated with potential hemodynamic instability during anaesthesia. Ciprofol introduces a cyclopropyl group to the chemical structure of propofol, forming an R-shaped hand structure and is characterised by rapid induction, rapid recovery, good controllability and a high degree of clinical safety.

**Methods:**

This prospective randomised, double-blind, controlled clinical trial aimed to assess the effects of ciprofol on haemodynamics and its safety and efficacy under general anaesthesia during thoracoscopic surgery. A total of 60 patients undergoing thoracoscopic surgery at First Affiliated Hospital of Guangxi Medical University between March 2023 and June 2023 were enrolled and 1:1 randomly assigned to receive anaesthesia with ciprofol or propofol. The primary outcomes were the incidences of cardiovascular events including hypertension, hypotension, bradycardia and tachycardia, the fluctuations in haemodynamic parameters. The secondary outcomes were injection pain, the bispectral index (BIS), the time of loss of consciousness and the time of disappearance of the eyelash reflex. For baseline characteristics, continuous variables were compared using Student’s t-tests or Wilcoxon rank-sum tests, while categorical variables were analysed using the Chi-square test. For fluctuations in haemodynamic parameters, repeated measures analysis of variance (ANOVA) was performed.

**Results:**

The Chi-square tests revealed no difference in the incidence of cardiovascular events (hypertension, hypotension, bradycardia and tachycardia) between ciprofol group and propofol group during both anaesthesia induction and maintenance. The ANOVA test showed that the decrease of mean arterial pressure (MAP) at T1 was gentler in the ciprofol group compared to the propofol group (*p* = 0.02). The difference between the heart rate at T5 and baseline (▲HR) in the ciprofol group was significantly lower than in the propofol group (*p* = 0.01). The ciprofol group had a lower incidence of injection pain in comparison with the propofol group (10.0% versus 23.3%, *p* = 0.028). The time of disappearance of the eyelash reflex was less in the ciprofol group than in the propofol group (*p* = 0.004).

**Conclusions:**

Ciprofol is a safe and effective anaesthetic that may be used as a substitute for propofol in the induction and maintenance of anaesthesia in thoracoscopic surgery.

**Trial registration:**

This study was registered in the Chinese Clinical Trial Registry (ChiCTR2300069650) on March 22, 2023.

**Supplementary Information:**

The online version contains supplementary material available at 10.1186/s12871-025-03054-6.

## Introduction

The main elements of enhanced recovery after surgery (ERAS) are the reduction of trauma and stress, including minimal surgical incisions and the rational use of general anaesthetic. Video-assisted thoracoscopic surgery (VATS) under general anaesthesia has been widely used in clinical lung surgery because it is of minimal trauma, thus allowing faster recovery [1, 2, 3]. Propofol is the most commonly used general anaesthetic due to its fast onset and rapid recovery properties [4, 5, 6], which facilitate early postoperative recovery. However, haemodynamic instability may occur during the induction and maintenance of propofol [7, 8], and injection pain is common [9, 10]. Ciprofol is a new type of intravenous anaesthetic that introduces a cyclopropyl group to form an R-shaped hand structure and increases the stereoscopic effect based on the chemical structure of propofol. Thus, it increases the affinity with the γ-aminobutyric acid type A (GABAA) receptor.

Ciprofol is characterised by rapid onset, a lower necessary dose, good controllability, a low incidence of adverse respiratory and circulatory events and less injection pain [11, 12, 13], greatly improving patient comfort during sedation. However, there is little literature on the impact of ciprofol on the haemodynamics of patients undergoing thoracoscopic surgery. Therefore, the aim of this study was to compare the safety and haemodynamic and anaesthetic effects of ciprofol and propofol on patients undergoing thoracoscopic surgery. We hypothesised that ciprofol as a sedative agent for patients undergoing thoracoscopic surgery would be safe and would result in more stable haemodynamics.

## Materials and methods

### Ethics statement

This study was conducted in accordance with the ethical standards established in the 1964 Declaration of Helsinki and its later amendments. It was registered in the Chinese Clinical Trial Registry (ChiCTR2300069650) on March 22, 2023. Informed consent was obtained from all participants, and research approval was obtained from the ethics committee of the First Affiliated Hospital of Guangxi Medical University (ethics registration no. 2023-K197-01).

### Study design, patients and grouping

This randomised study selected consecutive patients who underwent thoracoscopic lobectomy, wedge resection or segmentectomy between March and June 2023 at the Anesthesia Surgery Center of the First Affiliated Hospital of Guangxi Medical University in Nanning, China. The inclusion criteria were (1) planned thoracoscopic pulmonary surgery, (2) being 18–60 years of age, (3) an American Society of Anesthesiologists (ASA) physical status classification grade of I–II and (4) a body mass index (BMI) of 18–30 kg/m^2^. The exclusion criteria were (1) allergies or contraindications to opioids, propofol, ciprofol or other components, (2) a desire to withdraw from the trial, (3) a hypertension level of 3 or higher and (4) severe hepatic and renal insufficiency.

The study was conducted in accordance with the Consolidated Standards of Reporting Trials Statement and the CONSORT guidelines. All patients, data collectors and data analysts were blinded to the group allocation. A total of 60 patients were randomly assigned to receive either anaesthesia with ciprofol or anaesthesia with propofol, with 30 patients in each group. The random number table method was used for random grouping, and the random numbers along with the corresponding anaesthesia schemes were sealed in an envelope. Prior to entering the operating room, a nurse anaesthesiologist who was not involved in the study opened the envelope and prepared the medication according to the predetermined protocols. The relevant data were recorded and then returned to the sealed envelope for storage.

### Anaesthesia protocol

Preoperative testing was conducted prior to surgery. A routine five-lead electrocardiogram (ECG) was performed to monitor heart rate (HR) and heart rhythm. Noninvasive blood pressure measurement was carried out, pulse oxygen saturation levels were determined and bispectral index (BIS) were ascertained. A radial artery catheter was inserted under local infiltration anaesthesia with lidocaine to continuously monitor arterial blood pressure via arterial sensors. The baseline HR, mean arterial pressure (MAP) and BIS scores were recorded. When there was no change in HR or MAP for at least five minutes, the procedure began.

Regarding the induction of anaesthesia, after pre-oxygenation, the ciprofol group received a total dose of 0.4 mg/kg of ciprofol (Liaoning Hiske Pharmaceutical Co. Ltd., approval no. H20200013) at a rate of 6 mg/kg/h, and the propofol group received a targeted controlled infusion (TCI) dose of propofol (Sichuan Guorui Pharmaceutical Co. Ltd., approval no. H20030114) of 2.5–4 µg/ml. Sufentanil (Yichang Renfu Pharmaceutical Co. Ltd., approval no. H20054171) was administered at a constant rate of 2.5 µg/kg/min, with a total dose of 0.5 µg/kg in both groups. A total dose of 0.2 mg/kg of cisatracurium (Zhejiang Xianju Pharmaceutical Co. Ltd., approval no. H20090202) was injected intravenously. Intubation and mechanical ventilation were performed after the BIS score decreased below 60 and muscle relaxation was sufficient. Once the tube passed through the glottis under direct vision, the stylet was removed and the tubes were rotated 90 degrees clockwisely (for the right tube) or counterclockwisely (for the left tube) so that the tip of the tube aligns with the bronchus. The tubes were propelled until the resistance was encountered and cannot be pushed further. A fiberoptic bronchoscope (2.8 mm in diameter) was used to comfirm and adjust the position and depth of the tube, then the tubes were secured in place. To maintain anaesthesia, a 1–1.5 mg/kg/h dose of ciprofol was administered to the ciprofol group, and the TCI concentration for the propofol group ranged from 2.5 to 3.5 µg/ml. Both groups were continuously administered 0.05–0.2 µg/kg/min of remifentanil (Yichang Renfu Pharmaceutical Co. Ltd., approval no. H20030197), 5–10 µg/kg/min of rocuronium (Guangdong Jiabo Pharmaceutical Co., Ltd., approval no. H20183109), and 0.2 mg/kg/h of remazolam (Jiangsu Hengrui Pharmaceutical Co., Ltd., approval no. H20190034) until the end of surgery. The BIS index remained in the range of 40–60, and partial pressure of end-tidal carbon dioxide (PetCO2) values were maintained in the range of 35–45 mmHg during surgery.

The occurrences of hypotension, hypertension (blood pressure 20% higher than baseline), bradycardia (HR < 60 beats/minute) and tachycardia (HR > 100 beats/minute) were recorded during induction and maintenance. Hypotension was considered to occur when the MAP was lower than 65 mmHg or decreased by 30% from baseline, and complement of blood volume or a dose of 50 µg of phenylephrine was administrated intravenously. Atropine was administered intravenously in a dose of 0.25–0.5 mg when severe bradycardia (HR < 45 beats/minute) occurred.

### Outcome endpoints

The HR, the MAP and the BIS scores were determined before induction (baseline, T0), three minutes after induction (T1), immediately before tracheal intubation (T2), one minute after endotracheal intubation (T3), five minutes after endotracheal intubation (T4), at the beginning of surgery (T5) and at the end of surgery (T6). ▲MAP was the difference between MAP at each time point and baseline. ▲HR was the difference between HR at each time point and baseline. The time from the onset of anaesthesia induction to loss of consciousness and the time it took for the eyelash reflex to disappear was recorded. The main outcomes were the incidences of hypotension, hypertension, bradycardia, tachycardia and the fluctuation in haemodynamic parameters (MAP, HR, ▲MAP and ▲HR). The secondary outcomes were injection pain, BIS score, the time it took to lose consciousness and the time of the disappearance of eyelash reflex.

### Statistical analysis

IBM SPSS software version 26 and R 4.1.0 software were used for the normality tests and analysis of variance. The statistical methods for analysing the data were chosen based on the specific type of data. Continuous numerical variables that followed a normal distribution were expressed as mean ± standard deviation (SD), and differences between the two groups were compared using Student’s t-tests. Continuous numerical variables that did not follow a normal distribution were expressed as the median (lower quartile, upper quartile), and inter-group comparisons were performed using the Wilcoxon rank-sum tests. Categorical variables were expressed as frequency and percentage, and differences between the two groups were compared using the Chi-square test. For fluctuations in the haemodynamic parameters between the two groups, an ANOVA test was performed to compare the difference. Statistical significance was defined as *p* < 0.05.

The sample size was estimated from preliminary trial studies and was based on an estimate of the number of patients expected to participate in the trial and the minimum number of patients required to evaluate the practical purposes of the trial. When the type I error was 0.01 (bilateral), the power of the test was 95%. The incidence of hypotension was 25% in the ciprofol group and 75% in the propofol group. There were no significant differences between the groups in terms of the incidence of hypertension, bradycardia or tachycardia.

**Fig. 1:**
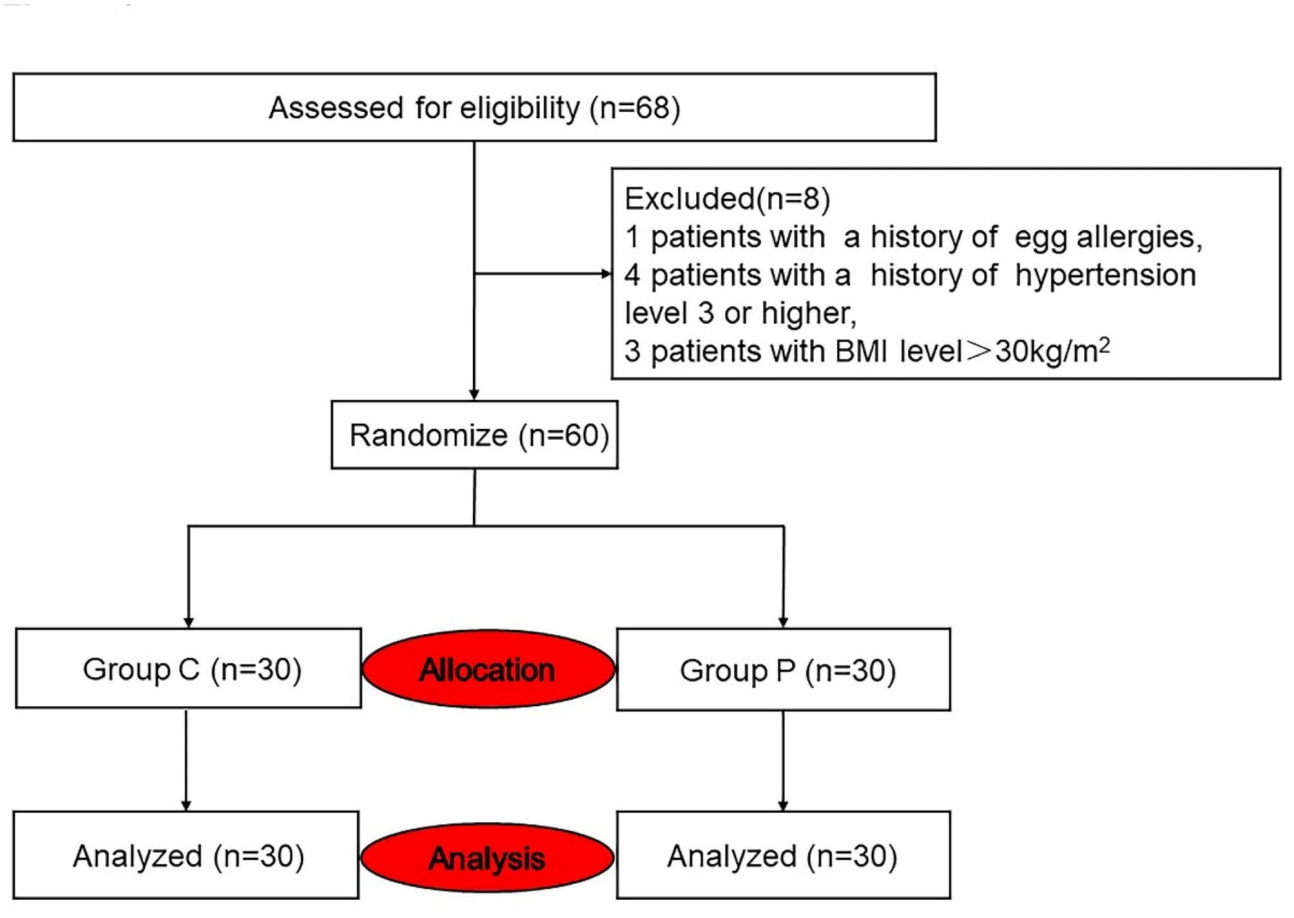
Consort flow diagram of patients C, ciprofol; P, propofol

## Results

Sixty patients (30 in each group) undergoing thoracoscopic lung surgery between March and June 2023 at the Anesthesia Surgery Center of the First Affiliated Hospital of Guangxi Medical University were included based on the inclusion and exclusion criteria (Fig. 1).

**Table 1:** Demographic characteristics of this study

Characteristics	Group *P*	Group C	*p* value
	*n* = 30	*n* = 30	
Sex^a^, n (%)			0.605
Female	17 (28.3)	15 (25.0)	
Male	13 (21.7)	15 (25.0)	
Age^b^ (years)	53 (43, 58)	53 (45.5, 57)	0.722
Body weight^c^ (kg)	61.47 ± 8.84	62.37 ± 9.88	0.711
Height^c^ (cm)	162.07 ± 7.35	163.07 ± 7.13	0.595
BMI^c^ (kg/m2)	23.38 ± 2.78	23.43 ± 3.22	0.940
Surgery			0.128
Pulmonary lobectomy^a^, n (%)	14 (23.3)	12 (20.0)	
Pulmonary wedging^a^, n (%)	11 (18.3)	17 (28.3)	
Segmentectomy^a^, n (%)	5 (8.3)	1 (1.7)	
Hypertension^a^, n (%)	4 (6.7)	4 (6.7)	1.000
Diabetes^a^, n (%)	2 (3.3)	1 (1.7)	1.000

C: ciprofol; P: propofol; BMI: body mass index. Data are expressed as mean ± standard deviation (SD) or the median (lower quartile, upper quartile) for continuous variables and number of participants (%) for categorical variables. ^a^*p*-value was obtained using Chi-square test; ^b^*p*-value was obtained using Wilcoxon rank-sum tests; ^c^*p*-value was obtained using Student’s t-tests

The baseline data are shown in Table 1. The total number of male patients was 28, of whom 15 were placed in the ciprofol group (25.0% of the study population) and 13 were placed in the propofol group (21.7% of the study population). The median ages were 53 (45.5–57) in the ciprofol group and 53 (43–58) in the propofol group, respectively. There were no significant differences between the two groups in terms of sex, age, height, weight, BMI or type of surgery.

**Table 2:** Incidence of intraoperative adverse events

Variable	Group *P*	Group C	*p* value
	*n* = 30	*n* = 30	
Anesthesia induction			
Injection pain, n (%)	14 (23.3)	6 (10.0)	0.028
Hypertension, n (%)	8 (13.3)	6 (10.0)	0.542
Hypotension, n (%)	20 (33.3)	14 (23.3)	0.118
Tachycardias, n (%)	9 (15.0)	4 (6.7)	0.117
Bradycardia, n (%)	8 (13.3)	8 (13.3)	1.000
Maintenance of anesthesia, n (%)			
Hypertension, n (%)	12 (20.0)	9 (15.0)	0.417
Hypotension, n (%)	12 (20.0)	10 (16.7)	0.592
Tachycardias, n (%)	4 (6.7)	4 (6.7)	1.000
Bradycardia, n (%)	10 (16.7)	14 (23.3)	0.292

C: ciprofol; P: propofol. Data are expressed as number of participants (%). *p*-value was obtained using Chi-square test

During anaesthesia induction, six patients (10.0%) experienced injection pain in the ciprofol group, which was significantly lower compared to the propofol group (14 patients, 23.3%) (*p* = 0.028). No significant differences were observed in terms of cardiovascular events (hypotension, hypertension, bradycardia or tachycardia) between the anaesthesia induction and maintenance periods (Table 2). In detail, during anaesthesia induction, the incidences of hypotension were 23.3% (Group C) and 33.3% (Group P), respectively. while the incidences of hypertension were 10.0% (Group C) and 13.3% (Group P). The incidences of bradycardia were identical in both groups with a percentage of 13.3%, whereas the incidences of tachycardia were 6.7% (Group C) and 15.0% (Group P), respectively. During anaesthesia maintenance, the incidences of hypotension were 16.7% (Group C) and 20.0% (Group P), and the incidences of hypertension were 15.0% (Group C) and 20.0% (Group P). The incidences of bradycardia were 23.3% (Group C) and 16.7% (Group P), while the incidences of tachycardia were 6.7% in both groups.

**Fig. 2:**
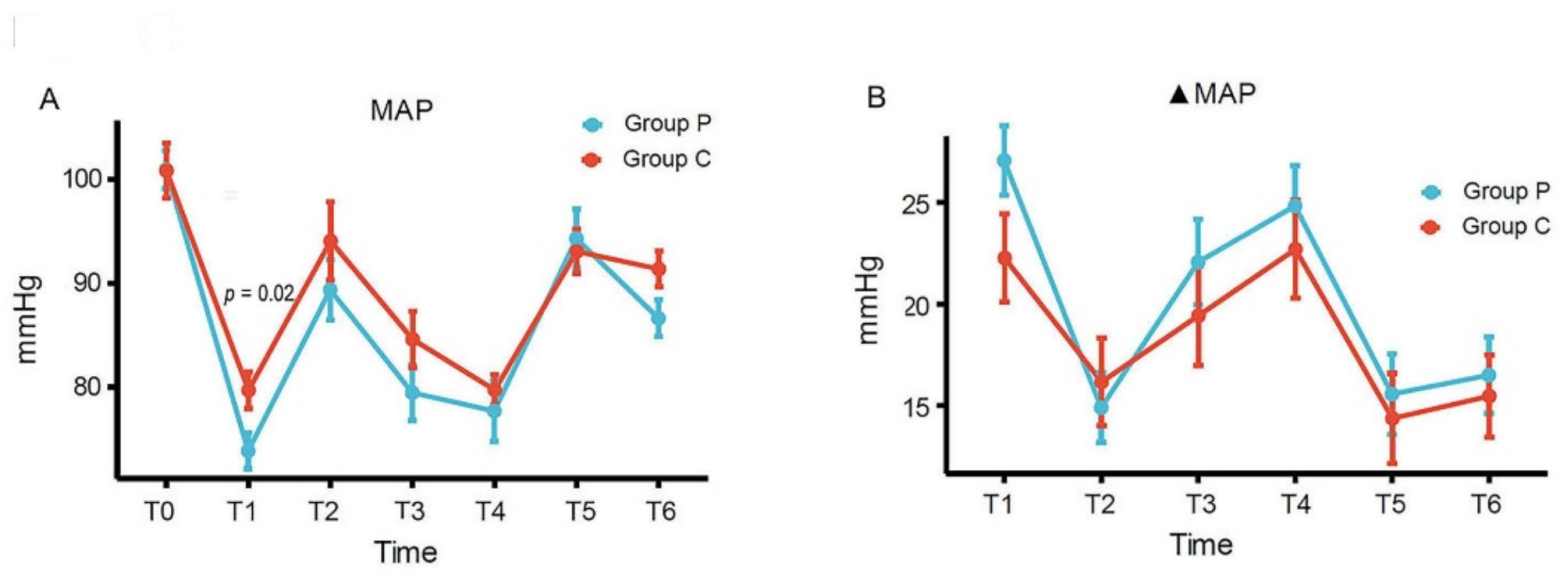
Fluctuations in blood pressure during anaesthesia C, ciprofol; P, propofol; MAP, mean arterial pressure; ▲MAP, difference between mean arterial pressure and baseline

**Fig. 3:**
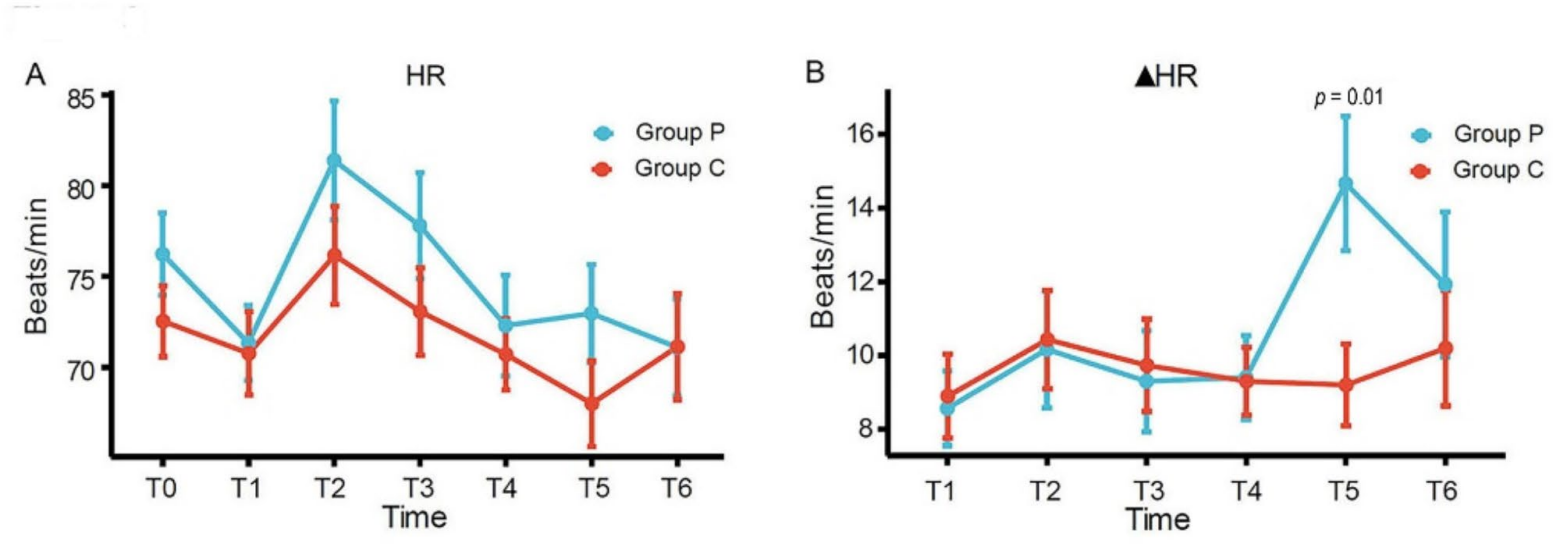
Fluctuations in heart rate during anaesthesia C, ciprofol; P, propofol; HR, heart rate; ▲HR, difference between heart rate and baseline

**Fig. 4:**
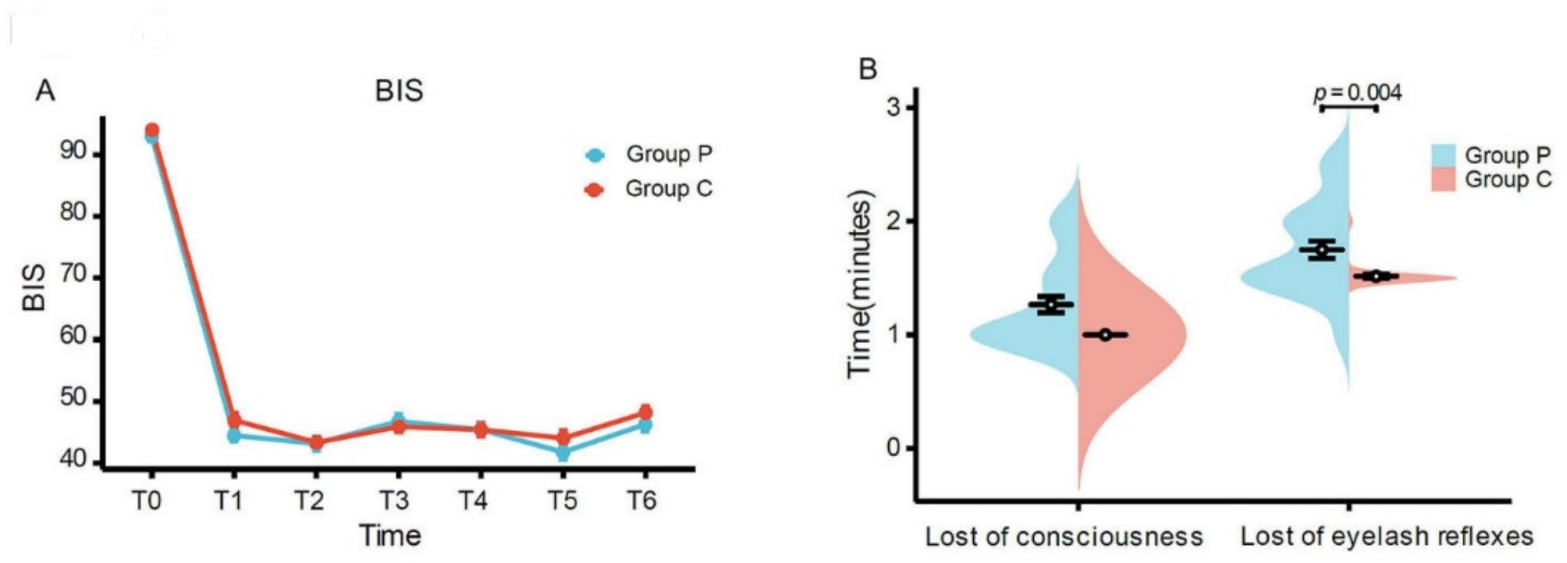
BIS, the time of loss of consciousness and eyelash reflex C, ciprofol; P, propofol; BIS, bispectral index

The blood pressure values declined significantly at three minutes (T1) after induction in both groups, but the decrease of mean arterial pressure (MAP) was gentler in the ciprofol group compared to the propofol group (Fig. 2A, *p* = 0.02). There was no significant difference in ▲MAP between the two groups (Fig. 2B). Compared to the propofol group, the ▲HR in the ciprofol group was smaller at T5, and the difference was significant (Fig. 3B, *p* = 0.01). The BIS score was not significantly different between the two groups at any time point (Fig. 4A). The time it took to lose eyelash reflex in the ciprofol group was less than that in the propofol group (Fig. 4B, *p* = 0.004).

## Discussion

In this randomised prospective clinical trial, we assessed the effects of ciprofol on haemodynamics during anaesthesia induction and maintenance in thoracoscopic surgery. We found that compared to the propofol group, the ciprofol group exhibited a similar incidence of cardiovascular complications (hypertension, hypotension, bradycardia and tachycardia), a significantly lower decline in MAP at three minutes after induction (T1), a lower ▲HR at the beginning of surgery (T5), a lower incidence of injection pain, and a shorter time to achieve loss of eyelash reflex after anaesthesia induction. Ciprofol displayed a high anaesthesia safety profile for the induction and maintenance of anaesthesia in thoracoscopic surgery.

Ciprofol and propofol have similar effects on the cardiovascular system, and both have cardiovascular inhibitory effects. Haemodynamic instability is a common adverse reaction to anaesthesia with propofol, with the incidence of intraoperative hypotension associated with propofol ranging from 25 to 67% [14, 15, 16]. This haemodynamic instability may result in damage to vital organs, such as the myocardium, kidneys and brain, which can lead to delayed recovery, cardiovascular or cerebrovascular problems, and an increase in perioperative mortality [17, 18]. This study observed a similar incidence of cardiovascular complications (hypertension, hypotension, bradycardia and tachycardia) between the two groups, indicating that ciprofol may be as safe as propofol for general anaesthesia. Consistent with our research, several previous studies indicated that the risk of hypotension associated with the use of ciprofol is similar to that of propofol [19, 20, 21]. However, some prior studies showed that ciprofol had a comparable or superior anaesthetic safety profile compared to propofol. For example, in a randomised trial involving 128 patients, Ji et al. [22] reported that the incidence of hypotension during gynaecological day surgery under general anaesthesia was similar between a ciprofol group and a propofol group. The overall occurrence of adverse events (including hypotension and bradycardia) was significantly lower with ciprofol anaesthesia compared to propofol anaesthesia (56.2% versus 92.2%, *p* <​ 0.05), which suggested a superior safety profile of ciprofol compared to propofol. This finding is corroborated by the results of another study, wherein Sun et al. [23] conducted a comprehensive meta-analysis of 12 randomised controlled trials (including 1,793 patients who underwent ciprofol sedation) and found a significantly lower incidence of hypotension in the ciprofol group compared to the propofol group (*p* = 0.02). These findings diverge from our study, one explanation is the disparities in study designs between this investigation and others, which may have resulted in variations in combination regimens (including other sedatives or analgesics). In addition, we noted that ciprofol and propofol led to the similar blood pressure fluctuation. A decline in blood pressure were observed in both groups three minutes after induction, following by an increase after intubation or incision and subsequent a gradual stabilization. The MAP at T1 was significantly higher in the ciprofol group compared to the propofol group, indicating that ciprofol possess a superior haemodynamic stability during the induction of general anaesthesia. Similarly, the change in ▲HR at T5 was significantly lower in the ciprofol group than in the propofol group, which suggested that ciprofol exhibited enhanced stability during surgical stimulation. These findings suggest that ciprofol may provide better maintenance of haemodynamic stability compared to propofol, consistent with the above-mentioned literature. The potential mechanism could be the enhanced binding affinity of ciprofol with the GABAA receptor, thereby resulting in increased efficacy. Consequently, lower doses of ciprofol could achieve similar effects to propofol in various clinical applications. Ciprofol is associated with less cardiovascular inhibition, a stabilizing effect on haemodynamics and a lower incidence of hypotension and other adverse reactions compared to propofol [24]. Therefore, ciprofol may be a more safe sedative than propofol [21].

Injection pain is among the most prevalent adverse reactions during the induction of propofol anaesthesia, with a reported incidence ranging from 50 to 80% [10, 25]. This could lead to haemodynamic instability, such as hypertension and tachycardia, which may impact a patient’s medical experience and comfort while under anaesthesia. Interestingly, our study revealed that ciprofol caused less pain during injection than propofol (10.0% versus 23.3%, *p* = 0.028). This phenomenon might be attributed to the particular cyclopropyl structure of ciprofol, which has a more stable spatial configuration and therefore lower lipophilicity than propofol. Consequently, the water-phase concentration of its emulsion is relatively low [26, 27].

The BIS score was 40–60 in the ciprofol group during the anaesthesia maintenance period, being similar to the score in the propofol group. This phenomenon could be ascribed to their similar molecular structure, in vivo absorption, distribution and metabolism and excretion rules. However, the time to lose eyelash reflex in the anaesthesia induction phase in the ciprofol group was less than that in the propofol group. This might be attributed to the cyclopropyl structure of ciprofol, which forms a chiral configuration that enhances its stereoscopic effect and thus increases its affinity with the GABAA receptor. Therefore, the effect of ciprofol is rapid. It follows that ciprofol has rapid onset and excellent anaesthetic efficacy in thoracoscopic surgery [28]. Moreover, the BIS score can be used to guide the depth of anaesthesia during ciprofol anaesthesia [20].

The present study has some limitations. This was a single-center prospective study with a small clinical sample. Thus, further multi-center and large-sample clinical studies are needed to confirm the results. In addition, the study focused only on anaesthesia for thoracoscopic surgery. Finally, it should be noted that the potential influence of vasopressor administration and fluid management on the incidence of hypotensive episodes was not systematically documented in the current study. This limitation underscores the need for future investigations employing standardised protocols to evaluate the potential impact of these interventions. Therefore, further studies are needed to investigate whether similar effects exist for other procedures.

## Conclusions

In conclusion, ciprofol is associated with more stable haemodynamics and less injection pain than propofol. It appears to be a safe and effective anaesthetic that can be used as a substitute for propofol in anaesthesia induction and maintenance in thoracic surgery.

## Electronic supplementary material

Below is the link to the electronic supplementary material.

Supplementary Material 1

## Data Availability

Due to the privacy regulations and limitations on the informed consent of participants, data cannot be openly accessible in public repositories. The data that support the findings of this study are available on request from corresponding author Jingchen Liu.

## Abbreviations

ASA: American Society of Anesthesiologists
BIS: Bispectral index
BMI: Body mass index
ECG: Electrocardiogram
ERAS: Enhanced recovery after surgery
GABAA: Gamma-aminobutyric acid type A
HR: Heart rate
▲HR: The difference between heart rate and baseline
MAP: Mean arterial pressure
▲MAP: The difference between mean arterial pressure and baseline
PetCO2: Partial pressure of end-tidal carbon dioxide
ANOVA: Repeated measures analysis of variance
SD: Standard deviation
TCI: Targeted controlled infusion
VATS: Video-assisted thoracoscopic surgery
